# Effect of the transcription factor YY1 on the development of pancreatic endocrine and exocrine tumors: a narrative review

**DOI:** 10.1186/s13578-021-00602-8

**Published:** 2021-05-13

**Authors:** Qun Chen, Wu-Jun Wang, Yun-Xuan Jia, Hao Yuan, Peng-Fei Wu, Wan-Li Ge, Ling-Dong Meng, Xu-Min Huang, Peng Shen, Tao-Yue Yang, Yi Miao, Jing-Jing Zhang, Kui-Rong Jiang

**Affiliations:** 1 Pancreas Center, The First Affiliated Hospital of Nanjing Medical University, 300 Guangzhou Road, Nanjing, 210029 Jiangsu People’s Republic of China; 2 Nanjing Medical University, Nanjing, China; 3 Jiangsu Province Hospital on Integration of Chinese and Western Medicine, Nanjing, China

**Keywords:** YY1, Pancreatic neuroendocrine tumor, Pancreatic ductal adenocarcinoma, Mutation, Tumor suppressor

## Abstract

Pancreatic tumors are classified into endocrine and exocrine types, and the clinical manifestations in patients are nonspecific. Most patients, especially those with pancreatic ductal adenocarcinoma (PDAC), have lost the opportunity to receive for the best treatment at the time of diagnosis. Although chemotherapy and radiotherapy have shown good therapeutic results in other tumors, their therapeutic effects on pancreatic tumors are minimal. A multifunctional transcription factor, Yin-Yang 1 (YY1) regulates the transcription of a variety of important genes and plays a significant role in diverse tumors. Studies have shown that targeting YY1 can improve the survival time of patients with tumors. In this review, we focused on the mechanism by which YY1 affects the occurrence and development of pancreatic tumors. We found that a YY1 mutation is specific for insulinomas and has a role in driving the degree of malignancy. In addition, changes in the circadian network are a key causative factor of PDAC. YY1 promotes pancreatic clock progression and induces malignant changes, but YY1 seems to act as a tumor suppressor in PDAC and affects many biological behaviors, such as proliferation, migration, apoptosis and metastasis. Our review summarizes the progress in understanding the role of YY1 in pancreatic endocrine and exocrine tumors and provides a reasonable assessment of the potential for therapeutic targeting of YY1 in pancreatic tumors.

## Background

As the second largest digestive gland of the human body, the pancreas is located behind the peritoneum and has both exocrine and endocrine functions [1, 2]. The exocrine pancreas comprises acinar cells and ductal cells, which produce a series of digestive enzymes that help the body catabolize fat and protein. The endocrine pancreas consists of islets comprising Langerhans cells, which secrete five hormones and play a major role in regulating blood glucose levels in humans [3, 4]. Pancreatic tumors—whether they affect exocrine glands, as does the most serious type, pancreatic ductal adenocarcinoma (PDAC), or endocrine glands, as do rare pancreatic neuroendocrine tumors (PNETs)—are highly difficult to treat, have a poor prognosis and urgently need to be addressed [5–7].

A PNET is a type of tumor derived from pancreatic multifunctional neuroendocrine stem cells that secrete various hormones and is also called an islet cell tumor [8, 9]. PNETs are rare tumors, accounting for less than 3% of all pancreatic tumors, but an upward trend in diagnosis has recently emerged owing to improved diagnostic techniques [10, 11]. PNETs are divided into nonfunctional and functional types. The former often lacks specific clinical manifestations and are detected only after a significant increase in tumor burden [12]. On the other hand, functional PNETs can be classified as insulinomas, gastrinomas, glucagonomas, somatostatinomas, etc., according to the clinical symptoms caused by the increase in the secretion of the corresponding hormone [13–16]. As a functional PNET, insulinoma is the most common cause of hypoglycemia caused by endogenous hyperinsulinemia [17, 18]. Clinically, most insulinomas are sporadic and often isolated, and they can be effectively cured by surgical resection. Surgical resection is the only curative therapy for PNETs. In another 10% of patients, the disease is hereditary; most of these patients harbor MEN1 gene mutations and often multiple mutations, with high malignancy [19–22]. Although insulinomas have inert growth behavior, most patients have distant metastases at diagnosis because of the lack of specific symptoms, and the prognosis is poor [23–26]. Therefore, the search for specific high-index indicators to predict sporadic insulinoma is important for the early diagnosis of PNETs. PDAC is the most common malignant pancreatic tumor, exhibiting a high invasion capability and a poor prognosis [26, 27]. It is the fourth most common cause of cancer-related death and the second most common cancer of the digestive tract [28]. Surgical resection is currently the only curative treatment for PDAC, but most patients who are diagnosed with PDAC either present with metastasis or have a tumor that is surrounded by critical blood vessels and are thus unable to undergo surgery [29–32]. In addition, PDAC does not respond to radiotherapy or chemotherapy, especially gemcitabine [23, 33–36]. The 5-year survival rate of patients with PDAC is only 8.2% [37]. Recent studies have shown that the pathogenesis of PDAC may be related to the activation of oncogenes and the inactivation of tumor suppressor genes [38–43]. Therefore, finding suitable indicators that can be used to screen high-risk populations and for the early detection of PDAC or PDAC precursors is particularly important for the prevention and treatment of PDAC.

Yin-Yang 1 (YY1) is a ubiquitous transcription factor of the GLI-Kruppel family, and it is a DNA-binding protein involved in a variety of biological functions, including cell growth, embryonic development, transcriptional regulation, large-scale chromosomal dynamics, and angiogenesis [44–49]. YY1 contains four C2H2-type zinc finger motifs with two specific domain components, as indicated by the terms yin and yang; these components can act as transcriptional repressors or activators [50, 51]. YY1 is located on the telomere region of chromosome 14 at segment q32.2 and regulates approximately 10% of the total human genome [52, 53]. YY1 is closely related to tumorigenesis. Regarding pancreatic tumors, Cao et al. confirmed a YY1 mutation in 30% of sporadic insulinomas, and patients with mutations have an earlier onset of insulinoma than patients without mutations [54]. In addition, YY1 has special importance in PDAC. Zhang et al. found that YY1 is highly expressed in PDAC, but the higher its expression is, the better the prognosis of the patient. Finally, this group found that YY1 acts as a tumor suppressor by downregulating MMP10 expression through the Muc4/ErbB2/p38/MEF2C axis [55]. Previous studies have elucidated that circadian rhythm disorders with various origins, such as genetic changes and metabolic abnormalities, are an important cause of malignant tumors [56, 57]. The pancreatic clock refers to the regular circadian rhythm of pancreatic exocrine and endocrine cells, which is the basis for the regulation of their normal biological processes [58, 59]. Interestingly, Jiang et al. reported that YY1 expression in the pancreatic epithelium normally follows a circadian rhythm and that high YY1 expression likely promotes malignant transformation of the pancreatic epithelium. These results suggested that YY1 plays an important role in the occurrence of PDAC [60].

Numerous studies have shown that YY1 is critical for the development and progression of pancreatic tumors. By reviewing and discussing the potential mechanism of action of YY1 in pancreatic tumors, we provide a reasonable perspective for YY1 as a therapeutic target for pancreatic tumors.

## Role of YY1 in PNETs

PNETs are rare endocrine tumors that are divided into functional and nonfunctional types, and most are insulinomas [61]. Studies have shown that YY1 is closely related to the occurrence and development of PNETs. As early as 2013, Cao et al. performed Sanger sequencing and pyrosequencing on 113 insulinoma patients and found 78 individual cell mutations. Their results indicated that the p.T372R missense mutation arises from the substitution of a neutral threonine with a positively charged arginine [54]. This relapse mutation is located in the DNA-binding region, which is the third zinc finger region of YY1, and alters the DNA-binding transcriptional activity of YY1 [62, 63].

## Relationship between YY1 and pancreatic β cells

Insulin secretion is controlled by secretion from pancreatic β cells. Irshad et al. found that the YY1 T372R mutation upregulates adenylate cyclase 1 (ADCY1) and a Ca^2+^ channel subunit (CACNA2D2) and is involved in cAMP and Ca^2+^ signaling, which plays an important role in the secretion of insulin from pancreatic β cells. Among the affected factors, the CACNA2D2 gene encodes the α-2-δ-2 auxiliary (porosity) subunit of the high voltage-gated Ca^2+^ channel. Ca^2+^ is a key factor in controlling vesicle release, and an increase in CACNA2D2 expression caused by YY1 mutations increases the intracellular Ca^2+^ concentration, causing fusion of insulin-containing vesicles to fuse with the plasma membrane and thereby promoting insulin release. Further studies showed that nutrients in the gut cause the release of the peptide hormone glucagon-like peptide 1 (GLP-1) from intestinal epithelial cells. GLP-1 is an agonist of the G protein-coupled receptor GLP1R. GLP1R activates adenylate cyclase 6 (ADCY6) and adenylate cyclase 8 (ADCY8) via its Gα subunit to produce cAMP, which can bind to cAMP-GEFII to mediate insulin secretion. In addition, upregulation of ADCY1 expression upregulates the activity of the GLP-1 pathway. In summary, a positive feedback system is active in ectopic expression of neuronal ADCY1 that promotes insulin secretion in β cells. Ca^2+^ entry induces ADCY1 activity, which in turn promotes further Ca^2+^ entry, allowing the two pathways to interact [64]. Cao et al. believe that the YY1 T372R mutation also increases pancreatic β cell proliferation and that the mechanism may be related to enhanced transcriptional activity after the YY1 T372R mutation. YY1 can increase the expression of IDH3A, UCP2 and COL1A1 through the mTOR pathway, thus promoting an increase in insulin secretion [54]. Therefore, this YY1 mutation can increase the proliferation and number of β cells, thus suggesting a theoretical basis for PNETs.

Moreover, Wang et al. showed the presence of complex mutations in genes such as MEN1, H3F3A, KDM6A, and ATR in insulinoma, and the YY1 T372R mutation also plays an important role. However, Wang believed that this mutation does not affect the proliferation of β cells, and there was no significant change in β cell proliferation activity after the overexpression of either wild-type or mutant YY1 [65].

## Relationship between YY1 and the onset of insulinoma

Cao et al. found that the YY1 T372R mutation was associated with the age at onset of insulinoma patients; patients with YY1 mutation were significantly older at onset than patients with no mutations (56 years for patients with the wild-type YY1 gene versus 46 years for patients with YY1 mutation). Therefore, Cao et al. believe that the YY1 mutation is an oncogenic mutation [54]. Moreover, Cao et al. and Lichtenauer et al. believe that YY1 mutations are significantly more common in women than in men, but large-sample studies are still needed to confirm this finding [54, 66]. Although sex-induced mutations at specific sites in target genes are unlikely, different steroid hormones may cause different changes in endocrine function, leading to different clinical outcomes; however, the specific mechanisms remain to be clarified [67, 68]. In addition, the expression intensity of YY1 in malignant insulinoma and benign insulinoma is significantly different, and the expression intensity of YY1 and the benign and malignant properties of insulinoma are positively correlated. For example, the higher the levels of YY1 expression are, the higher is the malignant potential of insulinoma [69].

In addition, the YY1 T372R mutation is likely to be related to ethnicity. Cromer et al. reported that the frequency of the YY1 T372R mutations in 43 American and Swedish patients was 33% (14/43) [64]. The study sample of Cao et al. included Chinese insulinoma patients, and the determined mutation rate was 30% (34/113) [54]. Lichtenauer et al. analyzed 47 samples from German insulinoma patients and found that the frequency of the YY1 T372R mutation was 13% (6/47), significantly lower than that in the Chinese and Caucasian patient cohorts [66]. In addition, Irshad et al. screened patients with sporadic insulinoma in India for the YY1 T372R mutation and found none of the above mentioned missense mutations at YY1 codon 372 in any of the 17 (100%) islet tissues analyzed [70]. Therefore, differences in genetic makeup based on ethnicity may affect disease susceptibility.

In conclusion, the YY1 T372R mutation is often found in benign and malignant insulinomas but not in malignant PNETs that do not secrete insulin or in PDACs, suggesting that this mutation is specific for the diagnosis of insulinoma (Table 1).

**Table 1:** Correlation between mutation of YY1 mutation and PNETs

	Author	Country	Exist of mutation	Number	Mechanism	Reference
1	Cao	China	Yes	34/113 (30%)	mTOR/IDH3A/UDP2/COL1A1	[51]
2	Parekh	America	Yes	2/23 (8%)	Mutation of YY1 have no influence to proliferation of β cell	[54]
3	Cromer	America	Yes	14/43 (32%)	ADCY1/cAMP-GEFII CACNA2D2/Ca^2+^	[56]
4	Wang	America	Yes	–	Mutation of YY1 have no influence to proliferation of β cell	[57]
5	Lichtenauer	Germany	Yes	6/47 (13%)	Mutation of YY1 have no influence to proliferation of β cell	[58]
6	Irshad	India	No	0/17 (0%)	–	[60]

## Role of YY1 in PDAC

Accumulating evidence shows that YY1 plays an important role in many human tumors, and its association with PDAC has been gradually elucidated. Zhang et al. showed that the expression of YY1 was significantly higher in PDAC tissues than in adjacent nontumor tissues (by qRT-PCR; 87/108, 80.6%) and normal pancreatic tissues; the immunohistochemical (IHC) results were similar, but the specific proportions were not reported. The higher the expression of YY1 was, the better the tumor differentiation and the lower the tumor-node-metastasis (TNM) stage. A ROC curve value of 1.159 was defined as the cutoff for differential YY1 expression (area under the curve, 0.858; p < 0.001). The results showed that the prognosis of patients with relatively high expression of YY1 (36/108, 33.3%) was significantly better than that of patients with relatively low expression of YY1 (72/108, 66.7%) (p < 0.001) [55]. In addition, Peng et al. found by qRT-PCR that YY1 expression was significantly higher in PDAC tissues than in adjacent tissues (specific proportion unknown, p < 0.001) [71].

## Effect of YY1 on the proliferation of PDAC cells

Zhang et al. reported that YY1 inhibits PDAC cell proliferation and tumor growth by regulating the SOX2OT-SOX2 axis. SOX2OT is a long noncoding RNA (lncRNA) and is one of the major transcription factors regulating pluripotent stem cells. A luciferase activity assay showed that the SOX2OT intron region is involved in the transcriptional activation of SOX2. qRT-PCR revealed that the expression level of YY1 in PDAC tissues was negatively correlated with the expression levels of SOX2OT and SOX2 in PDAC tissues, and the same results were obtained at the cellular level. Prognostic analysis indicated that low expression levels of SOX2OT or SOX2 predict good outcomes in patients with PDAC. YY1 and the promoter region of SOX2OT were shown to directly interact and to be inactivated by electrophoretic mobility shift assay (EMSA) and chromatin immunoprecipitation (ChIP), respectively, in vivo and in vitro. These experiments combined with those of animal experiments showed that YY1 negatively regulates SOX2OT expression and inhibits SOX2 expression, thereby suppressing PDAC cell proliferation [72].

In addition, Liu et al. found that the ability of YY1 to inhibit PDAC cell proliferation is also associated with the CDKN3/MdM2/P53/P21 signaling pathway. CDKN3 is a member of the protein phosphatase family and plays an important role in cell cycle regulation. YY1 negatively regulates CDKN3 expression by directly binding to the promoter region of CDKN3, and the decrease in CDKN3 expression inhibits the formation of the MDM2-P53 complex, which in turn upregulates P21 expression. P21 is a cyclin-dependent kinase inhibitor that induces cell cycle arrest and ultimately inhibits the proliferation of PDAC cells. The results of Cell Counting Kit 8 (CCK8) and EdU incorporation assays indicated that the proliferation of YY1-overexpressing BXPC-3 and PANC-1 cells was significantly inhibited compared with that of normal cells. This conclusion was confirmed by cell cycle analysis [73]. In addition to the regulatory effects of YY1 on the above pathways, the regulatory effects of YY1 on cell proliferation in poorly differentiated neuroendocrine carcinoma (PDNC) are associated with apoptosis. Overexpression of YY1 promotes the apoptosis of PDAC cells via transcriptional activation of Bax, a proapoptotic protein that mediates mitochondrial apoptosis. YY1 upregulates Bax transcription, and Bax than translocates to the mitochondrial membrane, leading to cytochrome c release and caspase activation. The initiation of this cascade reaction leads to apoptosis and inhibits tumor growth, thus blocking cancer progression [74].

## Effect of YY1 on the metastasis of PDAC

Zhang et al. also found that the ability of YY1 to inhibit the invasion and metastasis of PDAC cells may be mediated by downregulation of MMP10 via a MUC4/ErbB2/P38/MEF2C-dependent mechanism. MMP10 is a secreted protease that degrades the extracellular matrix, and TIMP2 is an inhibitor of MMP10. The expression level of YY1 was negatively correlated with the expression level of MMP10 but positively correlated with the expression level of TIMP2. Therefore, YY1 was suggested to promote the invasion and metastasis of YY1-knockdown cells via modulation of intercellular adhesion genes, including upregulation of MMP10 and downregulation of TIMP2. This conclusion was also confirmed in vitro. Studies have shown that the molecular pathway by which YY1 downregulates MMP10 is associated with the MUC4/ErbB2/P38-MAPK signaling axis. YY1 inhibits MUC4 gene transcription and expression by binding to a suppressor element in the MUC4 promoter, resulting in decreased phosphorylation of downstream ErbB2 and phosphorylation of p38/MAPK [75]. This pattern indicates activation of the ErbB2 and p38/MAPK pathways after YY1 silencing, thereby resulting in suppressed invasion and metastasis of PDAC cells [55]. In addition, Yuan et al. found that YY1 can affect the migration and metastasis of PDAC cells through the KRAS/NF-κB/YY1/miR-489 signaling axis [71]. KRAS mutation is one of the most common mutation types in PDAC [76]. Another study showed that KRAS mutation can activate the transcription factor YY1 through inflammatory NF-κB signaling, subsequently inhibiting the expression of the tumor suppressor gene miR-489, which affects the ability of PDAC cells to migrate and metastasize [71]. Shrivastava et al. and Lee et al. have reported that YY1 is involved in the regulation of c-Myc expression while Napoli Claudio et al. and Vernon et al. showed that c-Myc is involved in the transcriptional regulation of YY1; thus, an interaction between YY1 and c-Myc may exist [77–81]. In osteosarcoma, the intrinsically highly expressed YY1 can bind to CBP, which in turn binds to c-Myc to downregulate YY1 acetylation and jointly decrease the transcriptional activity of the promoter of the integrin α3 subunit, thus promoting the malignant phenotype of osteosarcoma cells [82]. YY1 can inhibit the transcription of c-Myc by reducing the formation of c-Myc/Max dimers and thereby play an anticancer role via the YY1/c-Myc/miR-141 axis in nasopharyngeal carcinoma [83]. Casey et al. found that Myc can bind to the promoters of the immune checkpoints molecules CD47 and PD-L1 and that inactivation of Myc can downregulate the expression of both genes [84]. YY1 might also enhance the antitumor immune response through binding to c-Myc [85]. In summary, the interaction between YY1 and Myc proteins, especially c-Myc, has rarely been reported in the development and treatment of PDAC and is worthy of further in-depth study. Hisatsune et al. showed that overexpression of YY1 upregulates the transcriptional activity of the hamster Muc1 promoter in a dose-dependent manner and is involved in the regulation of tumor invasion and migration. Muc1 is a cell surface glycoprotein that is abundantly expressed in cancer cells, and it has been shown to be involved in tumor metastasis and promotion [86]. Recently, our group conducted research on the role of YY1 in regulating the metastatic ability of PDAC and showed that YY1 can negatively regulate the expression of tubulin polymerization-promoting protein (TPPP), while overexpression of TPPP can significantly promote the invasion, migration and angiogenesis of PDAC [87–89]. In addition, feline sarcoma-related receptor (FER), a member of the receptor tyrosine kinase family, was also found to be able to directly bind to YY1, thereby inhibiting the formation of the FER/STAT3 complex and phosphorylation of STAT3, which reduces the expression of MMP2 and decreases the metastatic ability of PDAC [90–93]. Finally, Ge et al. revealed that the YY1/miR-548t-5p/CXCL11 axis suppresses PDAC progression by inhibiting the EMT pathway [94–96].

## Effect of YY1 on the autophagy ability and metabolism of PDAC cells

Overexpression of YY1 increases the number of autophagosomes, while increased expression of miR-30a via YY1 knockdown reduces the number of autophagosomes. YY1 inhibits the expression of miR-30a, a microRNA (miRNA) that downregulates the autophagy-related genes ATG5 and Beclin 1 to promote autophagy in PDAC cells. In addition, miR-30a inhibits the expression of YY1 to form a negative feedback loop [97]. Yang et al. showed that autophagy plays a dual role in PDAC: it initially inhibits tumorigenesis but also supports late tumor growth [98]. To enhance their invasive and metastatic abilities, tumor cells increase their energy and biosynthesis requirements and undergo biosynthetic and metabolic reprogramming [99]. Recent studies have shown that YY1 is also involved in this reprogramming through mechanisms such as promoting GLUT3 transcription to improve the uptake of glucose by tumor cells, activating PGK1 and PKM2 (two important enzymes in the glycolytic pathway), and promoting the transcription of G6PD, the key enzyme in the pentose phosphate pathway [100].

## Effect of YY1 on the pancreatic clock

Jiang W et al. showed that YY1 is physiologically expressed in normal pancreatic endothelial cells and highly expressed in PDAC cells or tissues. YY1 can inhibit the expression of the core clock gene BMAL1 by promoting the transcription of miR-135b, resulting in loss of the normal rhythm of pancreatic endothelial cells with the potential for malignant transformation. Moreover, high expression of YY1 was found to be correlated with higher T stage and poorer histological grade (n = 55) and with gemcitabine resistance (n = 37), but no statistically significant correlation was found between YY1 expression and the overall survival (OS) and progression-free survival (PFS) rates of the patients (n = 37). In addition, the higher the expression of YY1, the worse was the prognosis of patients (TCGA database, n = 141) [60]. These clinical results suggest that YY1 appears to play a promotive role in PDAC and is associated with chemotherapeutic resistance, in contrast to the results of Zhang et al.

YY1 may play opposite roles in different tumors, similar to the meaning of its Chinese name—yin and yang are both opposite and unified and can be transformed into each other under certain conditions [101]. Jiang W et al. analyzed the relationship between YY1 and the prognosis of patients in clinical data, but the results of their study may be affected by the sample size. Their evidence related to higher expression of YY1 was inconsistent, indicating a worse prognosis than lower expression in different cohorts. In addition, Zhang J et al. studied only the progression of PDAC and did not address the value of YY1 in the occurrence of PDAC. YY1 also exerts a bidirectional effect in breast cancer, and although understanding the mechanism by which YY1 plays opposite roles in a specific type of cancer is difficult, whether the specificity of different YY1 interactors in different tumor stages determines the function of YY1 is unclear [102, 103].

## Effect of YY1 on diabetes

Diabetes is a well-known major risk factors for PDAC [104, 105]. However, some studies have shown that YY1 is closely related to the pathogenesis of diabetes. YY1 can reduce the incidence of diabetes by regulating the transcription of the CXCL12 gene. Both PARP-1 and YY1 are important novel regulators of CXCL12 gene transcription in rat pancreatic β cells. PARP-1 has an inhibitory effect and YY1 has a strong activating effect on CXCL12 transcription. In the early stages of oxidative stress, YY1 shows low affinity for the CXCL12 promoter while PARP-1 shows high affinity, and this difference leads to downregulation of CXCL12. In contrast, in later stages of oxidative stress and pancreatic β cell injury, YY1 is highly expressed and binds strongly to the CXCL12 promoter, and CXCL12 expression thus exceeds PARP-1 expression. CXCL12 was found to be a pre-β cell growth-stimulating factor, revealing its antidiabetic potential both in vitro and in vivo. CXCL12 inhibits apoptosis by activating the prosurvival kinase Akt via mechanisms such as upregulation of the antiapoptotic protein Bcl-2 and phosphorylation of the proapoptotic protein Bax, thereby stimulating the survival of islet β-cells; moreover, some people with the CXCL12-3'A variant experience an early onset of diabetes [106]. In addition, a study by Klöting confirmed that the YY1 gene is a protective gene for rat diabetes. These observations prompt us to speculate that YY1 can inhibit the development of PDAC by reducing the incidence of diabetes [107].

**Fig. 1:**
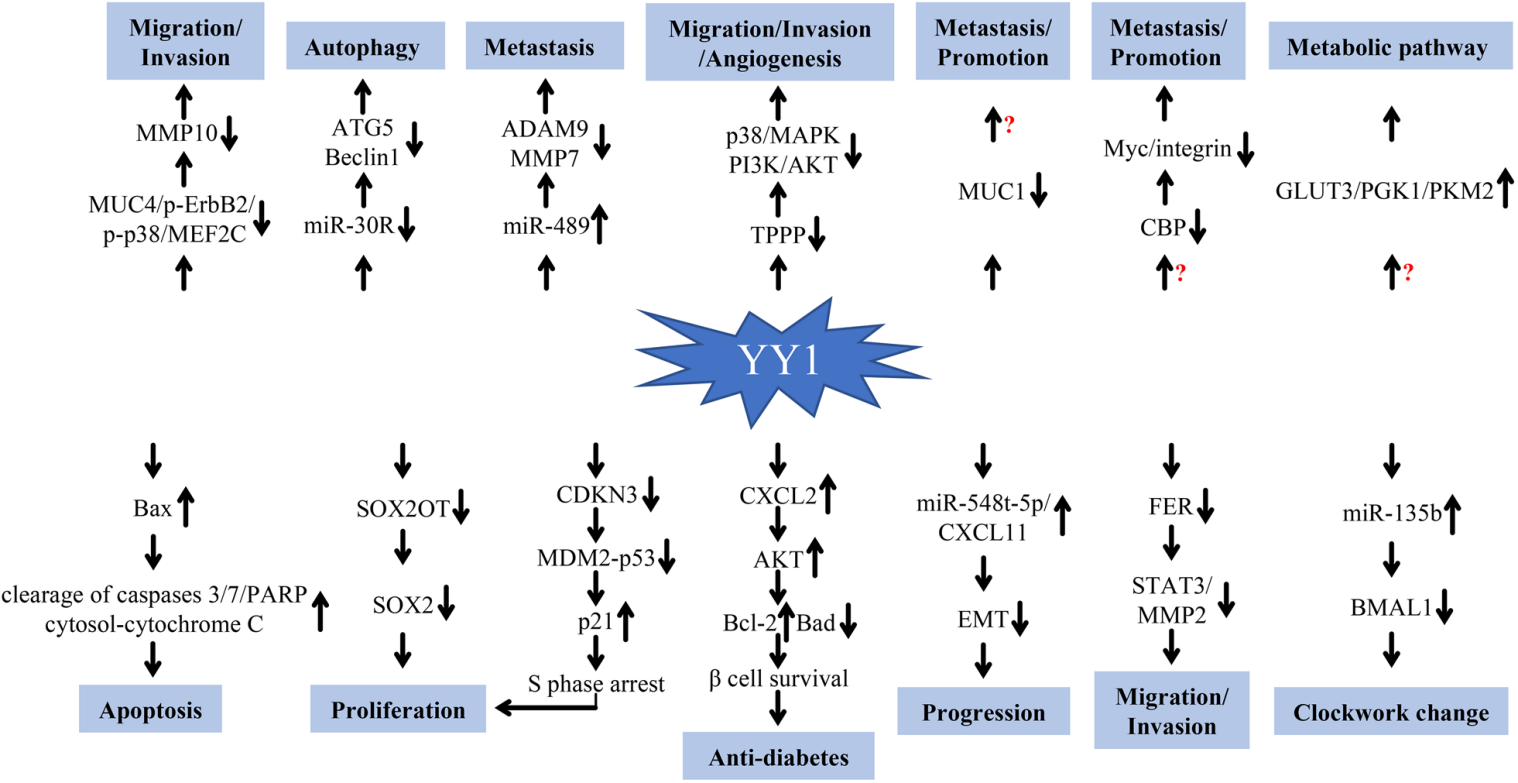
Relationships between YY1 and PDAC

Taken together, these results suggest that YY1 can affect the proliferation, invasion and migration of PDAC cells through various mediators, such as miRNAs, lncRNAs, CDKN3 and Bax (Fig. 1). The expression of YY1 is closely related to clinical characteristics. We speculated that increased expression of YY1 in pancreatic endothelial cells leads to malignant changes and promotes the occurrence of PDAC. However, after a PDAC tumor is formed, YY1 begins acting as a tumor suppressor, inhibiting its own high expression. The higher the expression of YY1, the greater is the inhibition of PDAC progression. This feedback loop might be a protective mechanism supplied by the human body.

## Discussion

We reviewed studies on YY1 and PNETs or PDAC and found that YY1 plays an extremely important role in pancreatic tumors. In insulinoma, although Irshad, et al. did not find the YY1 p.T372R heterozygous mutation in the Indian population, Cao, Cromer, and Wang, et al. confirmed that the mutation is present not only in the Chinese and the American populations but also in the German population. Moreover, the YY1 p.T372R mutation is a specific mutation that is different from mutations observed in pancreatic tumors that do not secrete insulin and in PDAC. Via further study, we demonstrated that YY1 mutation, which is associated with poor survival, may enhance the proliferation of β cells through the cAMP and Ca^2+^ signaling pathways. Insulinoma lacks specific clinical manifestations and may be easily missed. However, the YY1 p.T372R mutation can be used as an effective target for screening insulinoma patients and predicting prognosis [108, 109]. YY1 is a recently discovered pathogenic gene in insulinoma. Similar to its localization in PDAC cells, YY1 is localized mainly in the nucleus in insulinoma cells; however, YY1 is more highly expressed in malignant insulinoma than in PDAC and often indicates a worse prognosis.

The importance of YY1 in pancreatic tumors was further demonstrated in a study of YY1 and PDAC. PDAC is one of the deadliest cancers. According to data from the American Cancer Society, 44,430 deaths from PDAC occurred in 2018 [110, 111]. YY1 is regularly expressed in pancreatic epithelial cells, which could lead to dysregulation of the pancreatic clock and promote malignant transformation of epithelial cells. Zhang et al. found that YY1 is present in both the nucleus and cytoplasm of PDAC cells. YY1 is highly expressed in PDAC, indicating that YY1 may be a key factors involved in PDAC oncogenesis. However, YY1 acts as an inhibitor during the progression of PDAC, downregulating MMP10 expression via the MUC4/ErbB2/p38/MEF2C axis and downregulating ADAM9 and MMP7 expression via the KRAS/NF-kB/YY1/miR-489 axis. In addition, diabetes is an important pathogenic risk factor for PDAC. However, YY1 is also a transcription factor that restores pancreatic β cell function and inhibits the onset of diabetes [104, 112]. Although direct evidence is lacking, YY1 may also play an important role by affecting metabolic pathways in tumor cells. These results suggest that the transcription factor YY1 plays a crucial role in the occurrence and development of PDAC.

YY1 was found to impair the growth ability of embryos in a dose-dependent manner in a study using homologous recombination to generate knockout mice. In addition, complete ablation of YY1 can even lead to the arrest of cell division and the cell cycle, confirming the potential value of targeting YY1 in vivo [113]. Some drugs, such as betulinic acid, rituximab and galiximab, exert anticancer effects by downregulating YY1 expression by affecting the cannabinoid receptor or inhibiting the activity of NF-kB. [114–116]. YY1 may thus be a key signaling molecule mediating cancer progression [117]. To our knowledge, studies have shown that YY1 has a positive effect on improving chemotherapeutic tolerance through mechanisms such as regulation of EMT expression via the NF-kB/Snail/YY1/RKIP axis and regulation of Fas expression via the IFN-g/NO/NF-kB/YY1 axis. In addition, YY1 can inhibit autophagy in tumor cells via miRNAs, enhancing the sensitivity to chemotherapeutic drugs. YY1 also plays an important role in novel immunotherapies; it can interact with PD-L1 via p53, PI3K/AKT/mTOR, STAT3, and COX-2 to suppress tolerance to immunotherapy [51, 85, 118–122]. YY1 inhibits reactive oxygen species and promotes tumor cell death to suppress the immune system. Combination therapy with a YY1 inhibitor may enhance the sensitivity of tumor cells to immunotherapeutic drugs [123]. Encouragingly, YY1 has shown therapeutic effects in prostate cancer, hepatocellular carcinoma, breast cancer, cervical cancer, ovarian cancer, and multiple myeloma [124–129]. Therefore, a theoretical basis exists for treating PDAC by targeting YY1.

## Conclusion

In summary, since the discovery of YY1 in 1991 by Shi et al. and Park and Atchison, we have made significant progress in understanding YY1 and pancreatic tumors, including insulinomas and PDACs. Further study of the molecular mechanisms by which YY1 interacts with all possible binding proteins in different biological processes will faciliate in the development of new therapeutic approaches for pancreatic tumors.

## Data Availability

Not applicable.

## Abbreviations

YY1: Yin-Yang 1
PDAC: Pancreatic ductal adenocarcinoma
PNETs: Pancreatic neuroendocrine tumors
ADCY1: Adenylate cyclase 1
CACNA2D2: Ca2+ channels
GLP-1: Glucagon-like peptide 1
ADCY6: Adenylate cyclase 6
ADCY8: Adenylate cyclase 8
IHC: Immunohistochemistry
TNM: Tumor-node-metastasis
lncRNA: Long noncoding RNA
EMSA: Electrophoretic mobility shift assay
ChIP: Chromatin immunoprecipitation
CCK8: Cell counting kit 8
PDNC: Poorly differentiated neuroendocrine carcinoma
TPPP: Tubulin polymerization-promoting protein
FER: Feline Sarcoma-related
miRNA: MicroRNA
OS: Overall survival
PFS: Progression-free survival
